# Transitioning to sustainable dietary patterns: learnings from animal-based and plant-based dietary patterns in French Canadian adults

**DOI:** 10.3389/fnut.2023.1148137

**Published:** 2023-04-17

**Authors:** Gabrielle Rochefort, Didier Brassard, Sophie Desroches, Julie Robitaille, Simone Lemieux, Véronique Provencher, Benoît Lamarche

**Affiliations:** ^1^Centre Nutrition, Santé et Société (NUTRISS), Institut sur la Nutrition et les Aliments Fonctionnels (INAF), Université Laval, Québec, QC, Canada; ^2^École de Nutrition, Faculté des Sciences de l’Agriculture et de l’Alimentation, Université Laval, Québec, QC, Canada

**Keywords:** animal-based protein, plant-based protein, dietary pattern, diet quality, diet cost, healthy eating food index (HEFI)-2019, sustainability, sustainable diet

## Abstract

**Introduction:**

Many dietary guidelines promote the substitution of animal proteins with plant-based proteins for health benefits but also to help transitioning toward more sustainable dietary patterns. The aim of this study was to examine the food and nutrient characteristics as well as the overall quality and costs of dietary patterns consistent with lower intakes of animal-based protein foods and with higher intakes of plant-based protein foods among French Canadian adults.

**Methods:**

Dietary intake data, evaluated with 24 h recalls, from 1,147 French-speaking adults of the PRÉDicteurs Individuels, Sociaux et Environnementaux (PREDISE) study conducted between 2015 and 2017 in Québec were used. Usual dietary intakes and diet costs were estimated with the National Cancer Institute’s multivariate method. Consumption of animal- and plant-based protein foods was classified into quarters (Q) and differences in food and nutrient intakes, Healthy Eating Food Index (HEFI)-2019 scores and diet costs across quarters were assessed using linear regression models adjusted for age and sex.

**Results:**

Participants with lower intakes of animal-based protein foods (Q1 vs. Q4) had a higher HEFI-2019 total score (+4.0 pts, 95% CI, 0.9 to 7.1) and lower daily diet costs (-1.9 $CAD, 95% CI, –2.6 to -1.2). Participants with higher intakes of plant-based protein foods (Q4 vs. Q1) had a higher HEFI-2019 total score (+14.6 pts, 95% CI, 12.4 to 16.9) but no difference in daily diet costs (0.0$CAD, 95% CI, -0.7 to 0.7).

**Discussion:**

In a perspective of diet sustainability, results from this study among French-speaking Canadian adults suggest that a shift toward a dietary pattern focused primarily on lower amounts of animal-based protein foods may be associated with a better diet quality at lower costs. On the other hand, transitioning to a dietary pattern focused primarily on higher amounts of plant-based protein foods may further improve the diet quality at no additional cost.

## 1. Introduction

Current global food production has a major impact on the environment by contributing to 19–29% of total greenhouse gas emissions, land degradation and biodiversity loss (1, 2). A shift to more sustainable food production and consumption practices is therefore necessary to achieve the United Nations 2030 Sustainable Development Goals (3). As production of plant-based foods is less resource-intensive than production of animal-based foods (4), replacing the latter with the former has become one of the cornerstones of the sustainable diet paradigm (5–7). In this regard, the healthy and sustainable diet proposed in 2019 by the EAT-Lancet Commission advocates for a reduction in the global consumption of animal-based foods and an increase in the consumption of plant-based foods, including plant-based protein foods, so that efforts to feed the world’s population remain within the planetary boundaries (8). The Canada’s Food Guide (CFG)-2019 (9) also recognizes the impact of food choices on the environment, something that the Dietary Guidelines for Americans (DGA) 2020–2025 have yet to do (10).

In addition to pressuring the environment, intake of animal-based protein foods, especially red and processed meats, has been associated with unfavorable health outcomes in many studies. For example, consumption of red and processed meats has been associated with greater risks of type 2 diabetes, colorectal cancer, cardiovascular mortality and all-cause mortality (11–13). By contrast, intake of plant-based protein foods such as legumes, soy and nuts has been associated with lower total cholesterol, LDL cholesterol and triglycerides (14) as well as with lower risks of mortality (12, 15). Consequently, the health benefits of consuming plant-based protein foods are recognized in both the CFG-2019 (16) and the DGA 2020–2025 (10).

Animal-based protein foods represent a large share of the plate of typical dietary patterns in North America. For example, it has been estimated that two-thirds of the protein intake among Canadian adults came from animal-based foods in 2015 (17). This implies that transitioning the population’s intake from animal- to plant-based protein foods represents a sizeable task. Moreover, a decrease in the intake of animal-based protein foods may not be automatically compensated by an increase in the consumption of plant-based protein foods, and vice versa. In that context, a better understanding of the dietary patterns associated with lower intakes of animal-based protein foods and with higher intakes of plant-based protein foods is key to identifying the most feasible and acceptable dietary patterns consistent with health and diet sustainability in a given population. Thus, the aim of this study was to examine the food and nutrient characteristics as well as the overall quality and costs of dietary patterns consistent with lower intakes of animal-based protein foods and with higher intakes of plant-based protein foods among French Canadian adults. We hypothesized that a dietary pattern comprising more plant-based protein foods is associated more strongly with overall diet quality than a dietary pattern comprising less animal-based protein foods.

## 2. Materials and methods

### 2.1. Study design and population

Data from the web-based multicenter cross-sectional PRÉDicteurs Individuels, Sociaux et Environnementaux (PREDISE) study, which aimed to document associations between individual, social and environmental factors, and the adherence to dietary guidelines, were used for these analyses. Recruitment and complete procedures of the PREDISE study have been previously described (18). In short, between August 2015 and April 2017, participants aged 18–65 years from five different administrative regions of the Province of Québec (i.e., Capitale-Nationale/Chaudière-Appalaches, Estrie, Mauricie, Montreal, and Saguenay-Lac-St-Jean) were recruited through the services of a survey firm. Stratified sampling was used to obtain an age- and sex-representative sample of French-speaking adults from each of these five administrative regions. To be eligible, participants needed to have Internet access to complete the questionnaires. Exclusion criteria were pregnancy and lactation. Participants had a 3 weeks period to complete online questionnaires on sociodemographic characteristics and web-based 24 h dietary recalls (R24W). Afterward, participants were invited to an in-person visit at a research center where anthropometric measurements (i.e., height and weight) were taken. A total of 1849 participants met the inclusion criteria and gave their written consent and, among those, 1,147 completed at least one 24 h recall and were included in the study sample. The project was conducted in accordance with the Declaration of Helsinki and was approved by the Research Ethics Committees of Université Laval (ethics number: 2014-271), Centre hospitalier universitaire de Sherbrooke (ethics number: MP-31-2015-997), Montreal Clinical Research Institute (ethics number: 2015-02), and Université du Québec à Trois-Rivières (ethics number: 15-2009-07.13).

### 2.2. Dietary intake assessment

Dietary intakes were evaluated using three unannounced R24W (19, 20) in which participants were asked to report all foods they consumed the day before in their prepared and cooked forms, where applicable, thus accounting for food or nutrient losses and moisture change due to preparation (18). Nutrient intake values from the R24W are derived from the Canadian Nutrient File 2015. Foods reported were classified into seven food categories corresponding to the broad categories of foods consumed and of public health interest: (1)- animal-based protein foods (including yogurts, cheeses and unsweetened milk), (2)- plant-based protein foods (including plant-based yogurts, fortified plant-based cheeses that contain sufficient proteins (i.e., not less than 25 g per 100 g or 15 g per 100 g for products intended to resemble fresh cheese) and unsweetened plant-based beverages that contained at least 2.5 g of proteins per 100 ml), (3)- vegetables and fruits, (4)- refined grains, (5)- whole grains, and finally foods not recommended in the CFG-2019, which were further divided into (6)- processed meats and (7)- other foods group (see Supplementary Table 1 for further description of the food group classification). Consumption of foods in each food group was expressed in reference amount (RA) per 2,500 kcal. One reference amount corresponds to the portion size of one typical serving of each food in Canada (21). Nutrients consumption was expressed either in percentage of energy intake or in mg per 2,500 kcal.

### 2.3. Healthy eating food index-2019

Data from R24W were used to calculate the Healthy Eating Food Index (HEFI)-2019, which assesses the alignment of dietary patterns with recommendations on healthy food choices in the CFG-2019 (22, 23). The HEFI-2019 consists of 10 components: five that are based mostly on foods (Vegetables and fruits, Whole-grain foods, Grain foods ratio, Protein foods, Plant-based protein foods), one that is based on beverages (Beverages) and four that are based on nutrients (Fatty acids ratio, Saturated fats, Free sugars, Sodium). Scores for each component range from 5 to 20 points, and the HEFI-2019 total score has a maximum of 80 points (see Supplementary Table 2 for the HEFI-2019 components, points and scoring system). Higher HEFI-2019 scores reflect a greater adherence to recommendations on healthy food choices in the CFG-2019 and consequently, a better diet quality.

### 2.4. Daily diet costs

The daily diet cost was calculated for each participant and each 24 h recall by matching dietary recall data to a food price database created by our research team in collaboration with the Institut national de santé publique du Québec (INSPQ). The detailed methods for the creation of this food price database and the matching procedures have been described elsewhere (24). Briefly, in the R24W, each food reported is linked to a Bureau of Nutritional Science food group. A 2015–2016 Nielsen food price database was used to compute a standard price for each Bureau of Nutritional Science food group (*n* = 180) of the 2015 Canadian Nutrient File used in the R24W. This standard price was adjusted for material loss and for food preparation to account for moisture, fat loss and cooking gains. Then, the amount of each food or beverage reported in the R24W expressed in kilogram was multiplied by the corresponding Bureau of Nutritional Science food group price per kilogram for each 24 h recall and summed to obtain a daily diet cost. For the present study, the daily diet cost was adjusted to 2,500 kcal/day to estimate a cost for isocaloric dietary patterns and energy-adjusted daily diet costs were used in the statistical analyses.

### 2.5. Statistical analyses

#### 2.5.1. Estimating usual dietary intakes and diet costs

To account for within-individual random errors that affect dietary intakes measured with 24 h recalls, the National Cancer Institute (NCI)’s multivariate Markov Chain Monte Carlo method was used (25). This method allows the estimation of the distribution of usual (i.e., long term) food and nutrients intakes as well as of daily diet costs using regression calibration of data from repeated 24 h recalls. To better reflect variations in dietary intakes within individuals, the model was stratified by sex. The model included the following covariables: age and indicators for the sequence of 24 h recalls (i.e., first, second or third recall) and the day of the week (i.e., weekdays vs. weekend days including Friday). Certain foods were considered to be consumed episodically in the model if 10% or more of the population did not report consumption on the first dietary recall. Based on this criterion, the consumption of whole-grain foods, plant-based protein foods, processed meats and some beverages (i.e., sugary drinks, artificially sweetened beverages, vegetable and fruit juices, sweetened milk and plant-based beverages, alcohol, unsweetened milk and unsweetened plant-based beverages that are not a source of proteins) was considered episodic. All remaining foods and nutrients were considered to be consumed daily. The diet cost was also considered as a “daily” variable in the model. Estimated usual dietary intakes and costs among pseudo-individuals generated in the Monte Carlo simulation step of the multivariate method were pooled within each stratum. The HEFI-2019 total score and component scores were calculated from estimated usual intakes among pseudo-individuals.

#### 2.5.2. Descriptive statistics

SURVEY procedures were used when appropriate to account for the stratified design of the PREDISE study. To ensure sex- and age- representativeness in each administrative region, balancing weights were used since the final sample size of the PREDISE study was larger than originally planned. Consumption of animal- and plant-based protein foods, expressed in RA per 2,500 kcal, was first categorized into quarters based on raw intakes in the overall population. The distribution of sociodemographic variables across quarters of animal- and of plant-based protein foods consumption was estimated using the SURVEYFREQ procedure and differences were assessed with a chi-square test. Sociodemographic variables considered were sex (men and women), age (18 to <35, 35 to <49, 50–65 years), body mass index (BMI; normal < 25.0, overweight 25.0–29.9, obese ≥ 30), smoking status (never, former, occasionally, or daily), education (none/high school/trade, CEGEP, university) and household income (<30 000 $CAD, 30 000 to <60 000 $CAD, 60 000 to <90 000 $CAD, ≥90 000 $CAD).

#### 2.5.3. Association with food and nutrient intakes, diet quality, and diet costs

Linear regression models were used to examine the association between usual intakes of animal- or plant-based protein foods (independent variables, categorized as quarters) and usual intakes of other food groups and nutrients, HEFI-2019 scores and daily diet costs (dependent variables). For animal-based protein foods, quarter 4, which represents the group of participants with the highest consumption, was used as the reference. For plant-based protein foods, quarter 1, which represents the group of participants with the lowest consumption, was used as the reference. Models were adjusted for sex and age. Standard errors and 95% CI were estimated using 200 bootstrap resamples and normal approximation. All analyses were performed in SAS Studio (version 3.81 SAS Institute, Cary, NC, United States) and figures were generated in R Studio (version 2022.02.0; R Foundation for Statistical Computing, Boston, MA, United States).

## 3. Result

### 3.1. Characteristics of participants

As presented in Table 1, there was no major difference in the participants’ sociodemographic characteristics across quarters of animal-based protein food intake. In contrast, participants with the highest self-reported intake of plant-based protein foods were older, tended to have a lower BMI, were less likely to be occasional or daily smokers and had a higher education level than participants with lower intakes of plant-based protein foods (Table 2).

**TABLE 1:** Participants’ sociodemographic characteristics according to quarters of animal-based protein food intake^1^.

Characteristic (*n* = 1147)	Q4^2^ (>5.6–13.5) (%)	Q3 (>4.5–5.6) (%)	Q2 (>3.3–4.5) (%)	Q1 (0.0–3.3) (%)
**Sex**				
Female	26.8	26.9	24.1	22.1
Male	23.3	23.7	25.5	27.5
*p*	*0.11*			
**Age group**				
18–34 years	25.6	21.0	25.8	27.7
35–49 years	26.7	25.9	25.2	22.3
50–65 years	23.2	29.3	23.5	24.0
*P*	*0.16*			
**Body mass index group^3^**				
Normal (<25.0)	26.6	23.4	24.4	25.7
Overweight (25.0–29.9)	21.1	29.0	25.9	24.0
Obese (≥30.0)	26.5	24.8	24.1	24.6
*P*	*0.48*			
**Smoking**				
Never	26.3	23.9	25.6	24.2
Former	24.7	27.8	22.6	24.9
Occasional or daily	21.5	24.8	27.0	26.7
*P*	*0.67*			
**Education^3^**				
High school or less	24.7	21.9	27.1	26.3
CEGEP	23.8	25.1	28.8	22.4
University	25.5	27.5	21.4	25.6
*P*	*0.22*			
**Income^3^**				
<30 000 $CAD	22.1	22.7	25.6	29.6
30 000 to <60 000 $CAD	23.9	27.7	22.8	25.6
60 000 to <90 000 $CAD	28.7	23.2	24.6	23.5
≥90 000 $CAD	23.5	26.9	26.4	23.2
*P*	*0.68*			

^1^Values are percentages and will sum across columns. CAD, Canadian dollars; CEGEP, Collège d’Enseignement Général et Professionnel; Q, Quarter.

^2^Because these are only descriptive data, quarters of animal-based protein food intake were not identified based on usual dietary intakes obtained by the National Cancer Institute’s multivariate method. Range of animal-based protein food intake for each quarter is expressed as RA per 2,500 kcal.

^3^Body mass index group, *n* = 1,022 (125 missing values); Education, *n* = 1,087 (60 missing values); Income, *n* = 988 (159 missing values).

The italic values correspond to the *p*-value of the chi-square test.

**TABLE 2:** Participants’ sociodemographic characteristics according to quarters of plant-based protein food intake^1^.

Characteristic (*n* = 1147)	Q1^2^ (0.0–0.0) (%)	Q2 (>0.0–0.6) (%)	Q3 (>0.6–1.5) (%)	Q4 (>1.5–9.1) (%)
**Sex**				
Female	24.9	22.5	25.0	27.7
Male	29.4	23.1	25.4	22.1
*P*	*0.12*			
**Age group**				
18–34 years	31.6	24.2	21.9	22.3
35–49 years	26.0	24.2	24.9	24.9
50–65 years	23.6	20.1	28.8	27.5
*P*	*0.05*			
**Body mass index group^3^**				
Normal (<25.0)	21.9	22.7	25.4	30.1
Overweight (25.0–29.9)	27.3	21.6	27.9	23.2
Obese (≤30.0)	29.6	24.8	23.6	22.1
*P*	*0.08*			
**Smoking**				
Never	24.7	23.7	24.8	26.8
Former	25.9	21.3	26.7	26.1
Occasional or daily	38.9	22.8	23.2	15.0
*P*	*0.01*			
**Education^3^**				
High school or less	34.0	22.2	23.5	20.3
CEGEP	25.5	23.0	25.0	26.4
University	23.1	23.4	27.4	26.1
*P*	*0.06*			
**Income^3^**				
<30 000 $CAD	30.6	24.4	26.0	19.0
30 000 to <60 000 $CAD	29.6	19.2	26.3	24.9
60 000 to <90 000 $CAD	24.4	22.3	25.8	27.6
≥90 000 $CAD	20.6	26.3	27.5	25.6
*p*	*0.14*			

^1^Values are percentages and will sum across columns. CAD, Canadian dollars; CEGEP, Collège d’Enseignement Général et Professionnel; Q, Quarter.

^2^Because these are only descriptive data, quarters of plant-based protein food intake were not identified based on usual dietary intakes obtained by the National Cancer Institute’s multivariate method. Range of plant-based protein food intake for each quarter is expressed as RA per 2,500 kcal.

^3^Body mass index group, *n* = 1,022 (125 missing values); Education, *n* = 1,087 (60 missing values); Income, *n* = 988 (159 missing values).

The italic values correspond to the *p*-value of the chi-square test.

If the high plant-based protein food dietary pattern was the mirror of the low animal-based protein food dietary pattern and vice versa, then all (100%) participants would have been categorized into corresponding quarters of the two patterns. However, less than 30% of the entire cohort was categorized into corresponding quarters of usual plant-based and animal-based protein food consumption (Table 3), indicating a relatively strong mismatch between the two patterns.

**TABLE 3:** Proportion of all participants in each quarter of animal- and plant-based protein food intake^1,2^.

	Quarters of animal-based protein food intake				
		**Q4**	**Q3**	**Q2**	**Q1**
Quarters of plant-based protein food intake	Q1	8.4%	6.7%	5.5%	4.4%
	Q2	6.7%	6.5%	6.2%	5.6%
	Q3	5.6%	6.3%	6.6%	6.6%
	Q4	4.3%	5.5%	6.7%	8.4%

^1^Quarters of usual intake of animal- and plant-based protein foods are based on the National Cancer Institute’s multivariate method. Gray cells indicate group overlap.

^2^Q, Quarter.

### 3.2. Food and nutrient intakes

Table 4 presents the food and nutrient intakes across quarters of usual animal-based protein food intake. In this population, high intakes (Quarter 4) of animal-based protein foods corresponded to a mean of 6.5 RA/2,500 kcal (SE, 0.1) while low intakes (Quarter 1) corresponded to a mean of 2.8 RA/2,500 kcal (SE, 0.3). Participants with low compared to those with high intakes of animal-based protein foods (Quarter 1 vs. Quarter 4) had higher intakes of whole grains (+0.3 RA/2,500 kcal, 95% CI, 0.1 to 0.6), plant-based protein foods (+0.5 RA/2,500 kcal, 95% CI, 0.1 to 0.8), other foods not recommended in the CFG-2019 (+0.8 RA/2,500 kcal, 95% CI, 0.0 to 1.7), PUFA (+1.1%E, 95% CI, 0.6 to 1.5) and free sugars (+2.5%E, 95% CI, 1.2–3.8) as well as lower intakes of SFA (-2.1%E, 95% CI, -2.8 to -1.4) and sodium (-332 mg/2,500 kcal, 95% CI, -528 to -135).

**TABLE 4:** Usual food and nutrient intakes across quarters of animal-based protein food intake in French-speaking adults from Québec, Canada^1,4^.

	Q4 (Reference)	Q3	Q2	Q1
**Animal-based protein foods, RA/2,500 kcal**				
**Mean (SE)**	**6.5 (0.3)**	**4.8 (0.1)**	**3.9 (0.1)**	**2.8 (0.1)**
**Range**	**(>5.4–16.9)**	**(>4.3–5.4)**	**(>3.5–4.3)**	**(0.5–3.5)**
	**Mean (SE)^3^**	**Difference vs. Q4 (95% CI)^2,3^**		
Vegetables and fruits, RA/2,500 kcal	4.5 (0.2)	−0.0 (−0.3, 0.2)	0.0 (−0.4, 0.4)	0.0 (−0.6, 0.6)
Refined grains, RA/2,500 kcal	2.0 (0.1)	0.0 (−0.1, 0.1)	0.0 (−0.2, 0.2)	0.1 (−0.3, 0.4)
Whole grains, RA/2,500 kcal	1.2 (0.1)	0.1 (0.0, 0.2)	0.2 (0.0, 0.3)	0.3 (0.1, 0.6)
Plant-based protein foods, RA/2,500 kcal	0.8 (0.1)	0.1 (0.0, 0.2)	0.3 (0.1, 0.4)	0.5 (0.1, 0.8)
Processed meats, RA/2,500 kcal	0.6 (0.0)	−0.0 (−0.1, 0.0)	−0.1 (−0.2, 0.0)	−0.1 (−0.2, 0.0)
Other foods, RA/2,500 kcal	4.6 (0.2)	0.3 (0.1, 0.6)	0.5 (0.1, 1.0)	0.8 (0.0, 1.7)
MUFA, % energy intake	13.0 (0.2)	−0.1 (−0.4, 0.1)	−0.2 (−0.6, 0.2)	−0.3 (−1.0, 0.3)
PUFA, % energy intake	6.6 (0.1)	0.4 (0.3, 0.6)	0.7 (0.4, 0.9)	1.1 (0.6, 1.5)
SFA, % energy intake	12.9 (0.2)	−0.8 (−1.1, −0.4)	−1.3 (−1.8, −0.8)	−2.1 (−2.8, −1.4)
Free sugars, % energy intake	10.8 (0.4)	1.0 (0.5, 1.4)	1.6 (0.8, 2.4)	2.5 (1.2, 3.8)
Sodium, mg/2,500 kcal	3,671 (65.6)	−120 (−203, −36.2)	−206 (−337, −74.5)	−332 (−528, −135)

^1^Usual food and nutrient intakes are based on the National Cancer Institute’s multivariate method. All values were estimated using linear regression models adjusted for age and sex.

^2^Difference vs. the reference quarter (Q4) corresponds to the regression coefficient in the linear regression models (see section “2. Materials and methods”).

^3^SE and 95% CI are calculated using 200 bootstrap resamples.

^4^MUFA, monounsaturated fatty acids; PUFA, polyunsaturated fatty acids; Q, quarter; RA, reference amount; SFA, saturated fatty acids.

The food and nutrient intakes across quarters of usual plant-based protein food consumption is presented in Table 5. Low intakes (Quarter 1) of plant-based protein foods corresponded to a mean of 0.2 RA/2500kcal (SE, 0.0) while high intakes (Quarter 4) corresponded to a mean of 2.2 RA/2,500 kcal (SE, 0.1) in this population. Participants with high compared to low intakes of plant-based protein foods (Quarter 4 vs. Quarter 1) had higher intakes of vegetables and fruits (+1.8 RA/2,500 kcal, 95% CI, 1.2 to 2.4) and whole grains (+0.9 RA/2,500 kcal, 95% CI, 0.7 to 1.1), as well as lower intakes of refined grains (-0.5 RA/2,500 kcal, 95% CI, -0.8 to -0.2), animal-based protein foods (-0.8 RA/2,500 kcal, 95% CI, -1.4 to -0.2), processed meats (-0.4 RA/2,500 kcal, 95% CI, -0.5 to -0.2) and other foods not recommended in the CFG-2019 (-1.5 RA/2,500 kcal, 95% CI, -2.2 to -0.8). Participants with higher intakes of plant-based protein foods also had higher intakes of MUFA (+1.0%E, 95% CI, 0.5 to 1.6) and PUFA (+1.4%E, 95% CI, 0.9 to 1.8), and lower intakes of SFA (-1.5%E, 95% CI, -2.2 to -0.7), free sugars (-3.3%E, 95% CI, -4.6 to -2.0) and sodium (-256 mg/2,500 kcal, 95% CI, -460 to -52).

**TABLE 5:** Usual food and nutrient intakes across quarters of plant-based protein food intake in French-speaking adults from Québec, Canada^1,4^.

	Q1 (Reference)	Q2	Q3	Q4
**Plant-based protein foods, RA/2,500 kcal**				
**Mean (SE)**	**0.2 (0.0)**	**0.5 (0.0)**	**1.1 (0.1)**	**2.2 (0.1)**
**Range**	**(0.0–0.3)**	**(>0.3–0.8)**	**(>0.8–1.4)**	**(>1.4–11.7)**
	**Mean (SE)^3^**	**Difference vs. Q1 (95% CI)^2,3^**		
Vegetables and fruits, RA/2,500 kcal	3.6 (0.1)	0.7 (0.5, 0.9)	1.2 (0.9, 1.5)	1.8 (1.2, 2.4)
Refined grains, RA/2,500 kcal	2.3 (0.1)	−0.2 (−0.4, −0.1)	−0.4 (−0.6, −0.2)	−0.5 (−0.8, −0.2)
Whole grains, RA/2,500 kcal	0.9 (0.1)	0.3 (0.3, 0.4)	0.6 (0.4, 0.7)	0.9 (0.7, 1.1)
Animal-based protein foods, RA/2,500 kcal	4.9 (0.2)	−0.3 (−0.5, −0.1)	−0.5 (−0.9, −0.1)	−0.8 (−1.4, −0.2)
Processed meats, RA/2,500 kcal	0.7 (0.1)	−0.2 (−0.2, −0.1)	−0.3 (−0.4, −0.2)	−0.4 (−0.5, −0.2)
Other foods, RA/2,500 kcal	5.8 (0.2)	−0.6 (−0.9, −0.3)	−1.0 (−1.5, −0.5)	−1.5 (−2.2, −0.8)
MUFA, % energy intake	12.3 (0.2)	0.4 (0.1, 0.6)	0.6 (0.3, 1.0)	1.0 (0.5, 1.6)
PUFA, % energy intake	6.5 (0.1)	0.5 (0.3, 0.6)	0.8 (0.6, 1.1)	1.4 (0.9, 1.8)
SFA, % energy intake	12.6 (0.2)	−0.6 (−0.9, −0.3)	−0.9 (−1.4, −0.5)	−1.5 (−2.2, −0.7)
Free sugar, % energy intake	13.8 (0.4)	−1.3 (−1.9, −0.7)	−2.2 (−3.1, −1.3)	−3.3 (−4.6, −2.0)
Sodium, mg/2,500 kcal	3,648 (66.1)	−120 (−200, −39.1)	−187 (−316, −57.0)	−256 (−460, −52.4)

^1^Usual food and nutrient intakes are based on the National Cancer Institute’s multivariate method. All values were estimated using linear regression models adjusted for age and sex.

^2^Difference vs. the reference quarter corresponds to the regression coefficient in the linear regression models (see section “2. Materials and methods”).

^3^SE and 95% CI are calculated using 200 bootstrap resamples.

^4^MUFA, monounsaturated fatty acids; PUFA, polyunsaturated fatty acids; Q, quarter; RA, reference amount; SFA, saturated fatty acids.

### 3.3. HEFI-2019 scores and daily diet costs

**FIGURE 1:**
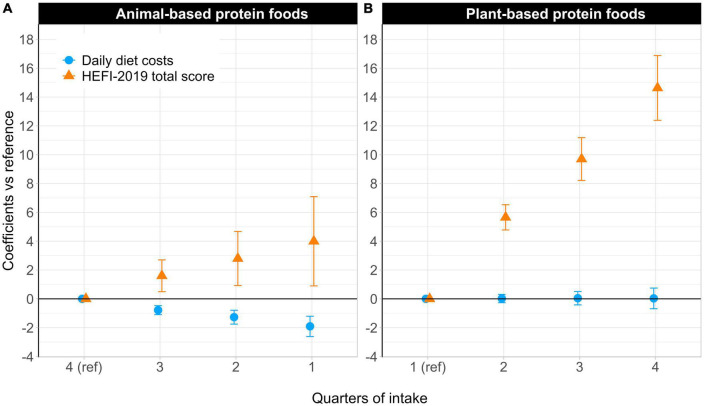
**(A)** Differences in the HEFI-2019 total score and daily diet costs across quarters of animal-based protein food intake. The HEFI-2019 total score and daily diet costs for the reference quarter (Q4) of animal-based protein food intake were 41.7 pts (SE, 0.8) and 13.7 $CAD per 2,500 kcal (SE, 0.2), respectively. **(B)** Differences in the HEFI-2019 total score and daily diet costs across quarters of plant-based protein food intake. The HEFI-2019 total score and daily diet costs for the reference quarter (Q1) of plant-based protein food intake were 36.3 pts (SE, 0.6) and 12.7 $CAD per 2,500 kcal (SE, 0.2), respectively. The regression coefficients scaled on the y-axis in both panels represent the differences in the HEFI-2019 total score (points) and daily diet costs ($CAD/2500 kcal) compared to the quarter of reference. Dietary intake data and costs standardized to 2,500 kcal were modeled using the National Cancer Institute’s multivariate method to reflect usual intakes. SE and 95% CI were calculated using 200 bootstrap resamples. HEFI-2019, Healthy Eating Food Index-2019; Ref, reference.

Differences in the HEFI-2019 total score and daily diet costs across quarters of animal- and plant-based protein food intake are presented in Figure 1. Participants with lower intakes of animal-based protein foods (Quarter 1 vs. Quarter 4) had a higher HEFI-2019 total score (+4.0 pts, 95% CI, 0.9 to 7.1) and lower daily diet costs (-1.9 $CAD, 95% CI, -2.6 to -1.2). Participants with higher intakes of plant-based protein foods (Quarter 4 vs. Quarter 1) also had a higher HEFI-2019 total score (+14.6 pts, 95% CI, 12.4 to 16.9) with no difference in daily diet costs (0.0 $CAD, 95% CI, -0.7 to 0.7). Differences in HEFI-2019 component scores between extreme quarters of animal-based protein food intake and of plant-based protein food intake are shown in Supplementary Figure 1.

## 4. Discussion

The aim of this study was to document the food and nutrient profiles as well as diet quality and costs of dietary patterns consistent with relatively low intakes of animal-based protein foods and with relatively high intakes of plant-based protein foods among French Canadians. Participants with low intakes of animal-based protein foods and participants with high intakes of plant-based protein foods in this population had relatively high intakes of whole grains, plant-based proteins foods and PUFA as well as low intakes of animal-based proteins foods, SFA and sodium. Furthermore, participants with high intakes of plant-based protein foods had high intakes of vegetables, fruits and MUFA and low intakes of refined grains, processed meats, other foods not recommended in the CFG-2019 and free sugars. Participants with low intakes of animal-based proteins foods had high intakes of other foods not recommended in the CFG-2019 and of free sugars. Finally, participants with low compared to high intakes of animal-based protein foods had a 4.0-point higher HEFI-2019 score and lower daily diet costs, while participant with high compared to low intakes of plant-based protein foods had a 14.6-point higher HEFI-2019 score with no difference in daily diet costs.

Findings from our study are partially consistent with previous studies comparing the dietary patterns of low vs. high meat-eaters. For example, a recent study in the Netherlands reported that intakes of nuts and seeds were higher in men and women with a lower consumption of meat (26). However, and unlike our own observations, Dutch participants with a relatively low meat consumption compared to those consuming more meat had higher intakes of vegetables and refined grains (26). In the United Kingdom, adults with low meat consumption were found to consume more whole grains, soy, legumes, nuts, seeds, vegetables and fruits, and less refined grains, fried foods, alcohol and sugar sweetened beverages compared to regular meat-eaters (27), partially confirming our observations regarding the dietary patterns of low vs. high animal-based protein food consumers.

Previous studies have also examined the dietary patterns associated with different intakes of plant-based proteins. Aggarwal and Drewnowski reported that a greater consumption of plant-based proteins was associated with higher intakes of fruits and vegetables, and with lower intakes of solid fats and added sugars, which is consistent with data from the present study (28). Along with a greater consumption of plant-based protein foods, vegetarians and vegans have also been reported to consume more fruits, vegetables and whole-grain foods, and less refined grains, processed meats and fried foods than regular meat eaters (29, 30), which is consistent with data from the present study. Finally, French-Canadian adults who reported consuming more plant-based protein foods also had higher intakes of PUFA and MUFA, and lower intakes of SFA and sodium than those who consumed little plant-based protein foods, consistent with previous findings (30, 31).

The literature suggests that healthy dietary patterns generally cost more than unhealthy dietary patterns, regardless of the diet quality index used, including the HEFI-2019 (24, 32–35). In the present study, participants with a relatively lower intake of animal-based protein foods (Quarter 1 vs. Quarter 4 in this population) had a better diet quality (+4.0 points in the HEFI-2019 total score) at lower daily diet costs. This agrees with a recent study having shown that the intake of animal proteins *per se* was negatively associated with diet quality and positively with diet costs (28). On the other hand, we found that participants with a diet characterized by higher vs. lower amounts of plant-based protein foods (Quarter 4 vs. Quarter 1) had a more pronounced difference in diet quality (+14.6 points in the HEFI-2019 total score) at no additional daily diet cost. This marked increase in the HEFI-2019 is consistent with the better diet quality associated with vegetarian diets (30, 36) and with the replacement of animal-based protein foods by plant-based protein foods (37). Moreover, diets with more energy from plant-based protein have been previously associated with a better diet quality with minimal increase in daily diet costs (28).

The present findings provide perspectives on the differences in dietary intakes and quality that may be expected as dietary recommendations increasingly advocate for a reduction in the consumption of animal-based protein foods and an increase in the consumption of plant-based protein foods. First, only a small proportion of participants were categorized into both the low quarter of animal-based protein food intake and the high quarter of plant-based protein food intake, indicating that these are quite distinct dietary patterns. The differences observed between high compared with low plant-based protein food dietary patterns suggest that high plant-based protein food consumers may be more prone to consider health or nutrition concerns when choosing foods, as observed among vegetarian adult populations (38, 39). Second, the quarter of the population with the highest consumption of plant-based protein foods, and with the highest HEFI-2019 score, still reported consuming approximately 4 RA (or “servings”) of animal-based protein foods per day. This indicates that a slight increase in the consumption of plant-based protein foods may be sufficient to observe a marked improvement in the overall quality of the diet of French Canadians at no additional cost, without a drastic reduction in the consumption of animal-based protein foods or even its exclusion from the diet. This supports the potential acceptability of adopting healthier and more sustainable protein-related dietary patterns in this population as it does not require major changes in the diet.

This study has several strengths including the use of an age- and sex-representative sample of French-speaking adults in each of the five pre-selected most populated administrative regions of the province of Quebec. Another strength is the use of the NCI multivariate method to account for random errors affecting dietary intake data measured by repeated 24 h recalls and thus, the ability to generate usual dietary intakes and daily diet costs rather than data on “any given day.” Characterizing dietary patterns based on both high/low animal- and plant-based protein food intake, rather than just one or the other, is original and another strength. The use of the HEFI-2019, a validated index reflecting adherence to the most recent recommendations on healthy food choices in Canada, as a proxy of diet quality is also a strength. Limitations also need to be addressed. First, the data used are from 2015 to 2017, which may not represent the current dietary patterns of French Canadians since data from industrialized countries suggest a slight but ongoing decrease in animal-based food intakes and an increase in plant-based food intakes in recent years (40, 41). Secondly, there are some limitations specific to the food price database used. For example, food prices were not available by type of store, season, geographic location, or other demographic factors, and do not represent the lowest price available. Moreover, by using Nielsen food price data, we assumed that all foods and beverages were bought from grocery or big box stores, and food waste was not considered. Thirdly, results cannot be generalized to all other populations given the relatively high education and income of the study sample. Finally, the lack of information on the environmental impact of the documented dietary patterns of French Canadians does not allow us to have a full overview of their sustainability.

In conclusion, data from this cohort of French-speaking Canadian adults suggest that a transition toward dietary patterns characterized by lower amounts of animal-based protein foods may reasonably improve diet quality at lower daily diet costs in this population. However, shifting to a diet with more plant-based protein foods may be even more effective to enhance diet quality at no additional cost. These data suggest that promoting the adoption of plant-based dietary patterns, without full exclusion of animal-based protein foods, is promising and should continue to be one of the key strategies in dietary guidelines to achieve healthier and more sustainable dietary patterns. Strong public health initiatives may be required to facilitate the adoption of such dietary patterns at the population level. Additional research on the environmental impact of dietary patterns with higher amounts of animal- or plant-based protein foods among French Canadians and in other populations is needed to better assess and compare their sustainability.

## Data availability statement

The original contributions presented in this study are included in the article/Supplementary material, further inquiries can be directed to the corresponding author.

## Ethics statement

This study involving human participants was reviewed and approved by Research Ethics Committees of Université Laval (ethics number: 2014-271), Centre Hospitalier Universitaire de Sherbrooke (ethics number: MP-31-2015-997), Montreal Clinical Research Institute (ethics number: 2015-02), and Université du Québec à Trois-Rivières (ethics number: 15-2009-07.13). The participants provided their written informed consent to participate in this study.

## Author contributions

GR, JR, VP, SD, SL, and BL designed research. GR and DB performed statistical analysis. GR and BL wrote the manuscript. BL had primary responsibility for final content. All authors read and approved the final manuscript.
