# Supplementary material for: Transitioning to sustainable dietary patterns: learnings from animal-based and plant-based dietary patterns in French Canadian adults
Source: Front Nutr. 2023 Apr 17;10:1148137. doi: 10.3389/fnut.2023.1148137 (PMC10150026; doi:10.3389/fnut.2023.1148137)
Supplement: Supplementary file 1 [file Data_Sheet_1.docx]

Supplementary Material

# Supplementary Tables

**Supplemental Table 1.** Food group classification^1^

| **Food group** | **Included foods** | **Description** |
| --- | --- | --- |
| 1-Animal-based protein foods (including unsweetened milk, yogurt and cheese)^2^ | Yogurts and kefir | Yogurt, yogurt drinks, kefir and similar products made from the milk of cows, goats and other animals. |
|  | Cheeses | All types of cheese made from the milk of animals such as cow, goat and buffalo cheese. Include cheese with ingredients such as herbs, flavourings, spices. |
|  | Other milk-based foods | All types of dairy-based desserts, soups and sauces. |
|  | Red meats | All beef, pork, lamb and goat meat from domesticated animals. |
|  | Game meats | Meat from all wild land and sea mammals. |
|  | Poultry and wild birds | Meat from all types of birds. |
|  | Eggs | All eggs. |
|  | Fish and shellfish | All fish and shellfish. |
|  | Organ meats | The organs of all wild and domesticated animals. |
|  | Unsweetened milk | All animal-based milks such as cow, goat or buffalo milk as well as milk-based beverages not artificially- or sugar-sweetened. Includes skim milk powder. |
| 2-Plant-based protein foods (including unsweetened plant-based beverages that contain sufficient protein)^3^ | Plant-based yogurts | Fortified Yogurt, yogurt drinks, kefir and similar products made from plant-based sources that contain protein. |
|  | Fortified plant-based cheeses (that contain sufficient protein) | All cheese-like products made from plant-based sources that contain sufficient protein. The protein content must be not less than 25g/100g. For products intended to resemble fresh cheese, the protein content must be not less than 15g/100g. |
|  | Legumes | Beans, dried peas, lentils, soybeans and tofu (excluding soy beverages). |
|  | Simulated meats | Simulated meat products such as veggie/meatless patties, nuggets. Foods are typically made with protein isolates. Simulated meats must have similar protein quality as the product they are simulating. |
|  | Nuts and seeds | All nuts and seeds, including peanuts. |
|  | Unsweetened plant-based beverages (that contain sufficient protein) | Plant-based beverages not artificially- or sugar-sweetened, including soy, that contain sufficient protein. The protein content must be not less than 2.5g/100mL. |
| 3-Vegetable and fruits | Fruits | All types of fruit (not including juice). Includes fruit purees. |
|  | Dark green vegetables | Dark leafy greens and other vegetables that are dark green in color. |
|  | Deep yellow or orange vegetables | Carrots, winter squash and other deep yellow or orange vegetables. |
|  | Starchy vegetables | Potato, white and yellow corn, sweet potato, yam, parsnip, plantain, cassava, bread fruit, taro root, burdock root and lotus root. |
|  | Other vegetables | All remaining vegetables that do not fit into any of the other vegetable subgroups. Includes tomato sauce. |
| 4-Refined grains | Non-whole grain and non-whole wheat foods | All refined grain products that do not have whole grain or whole wheat as the first ingredient. Includes cornmeal, tapioca and other plant-based starchy items. |
| 5-Whole grains | Whole grain, whole grain foods and whole wheat foods | All whole grains that are 100% whole grain and all grain foods with whole grain or whole wheat as the first ingredient. |
| 6- Processed meats | Processed meats | All meats preserved by smoking, curing salting or the addition of chemical preservatives. |
| 7- Other foods not recommended in the CFG-2019 | Other plant-based foods (that do not contain sufficient protein) | All types of plant-based foods that are intended for use as alternatives to milk-based foods and that do not contain sufficient protein. |
|  | Condiments, sauces and lower fat dressings | Condiments, sauces, pickled vegetables, lower fat dressings, fillings and seasonings with salt and sugar added, other foods with small reference amounts that accompany or add flavour to other foods. |
|  | Other snack foods | Chips, pretzels, extruded snacks. Includes sugar free versions of these snack foods. |
|  | High sugar and/or high fat foods | Higher fat and/or sugar foods where the first ingredient is either sugar or fat. |
|  | Canadian Nutrient File (CNF) recipes | CNF recipes only. These are mixed dishes that cannot be assigned a food grouping since they contain significant amounts of ingredients from different food groupings. In the CNF, ingredient proportions for recipes are not available so recipes cannot be taken apart. |
|  | Baby and toddler foods | Foods directed for consumption by infants and toddlers. |
|  | Meal replacements and supplements | Meal replacements and foods considered to be supplements. |

^1^This table is adapted from Health Canada, 2022 (1).

^2^ Artificially- or sugar-sweetened milks are not classified as animal-based protein foods in agreement with the CFG-2019 which does not consider them to be healthy beverages.

^3^ Artificially- or sugar-sweetened plant-based beverages are not classified as plant-based protein foods in agreement with the CFG-2019 which does not consider them to be healthy beverages.

**Supplemental Table 2.** Healthy Eating Food Index (HEFI)-2019 components, points and scoring system.^1^

|  | Component | Measurement | Maximum points | Unit | Standard for minimum score | Standard for maximum score |
| --- | --- | --- | --- | --- | --- | --- |
| 1 | Vegetables and fruits | Total vegetables and fruits/  Total foods | 20 | RA/RA | No vegetables and no fruits | ≥0.50 |
| 2 | Whole-grain foods | Total whole-grain foods / Total foods | 5 | RA/RA | No whole-grain foods | ≥0.25 |
| 3 | Grain foods ratio | Total whole-grain foods / Total grain foods | 5 | RA/RA | No whole-grain foods | 1.0 |
| 4 | Protein foods | Total protein foods /  Total foods | 5 | RA/RA | No protein foods | ≥0.25 |
| 5 | Plant-based protein  foods | Plant-based protein foods/  Total protein foods | 5 | RA/RA | No plant-based protein foods | >0.50 |
| 6 | Beverages | (Plain water including carbonated +  unsweetened beverages)/  Total beverages | 10 | g/g | No water and no unsweetened beverages | 1.0 |
| 7 | Fatty acids ratio | (Mono- + polyunsaturated fat)/  Total saturated fat | 5 | g/g | ≤1.1 | ≥2.6 |
| 8 | Saturated fats | Total saturated fat / energy | 5 | %E (kcal/kcal) | ≥15%E | <10%E |
| 9 | Free sugars | Total free sugars / energy | 10 | %E (kcal/kcal) | ≥20%E | <10%E |
| 10 | Sodium | Total sodium / energy | 10 | mg/kcal | ≥2.0 | <0.9 |
| - | Total HEFI-2019 | - | 80 | - | - | - |

^1^Adapted from Brassard et al. 2022 (2)

# Supplementary Figures

**Supplemental Figure 1. (A)** Radar plot of the HEFI-2019 component scores comparing Quarter 1 (yellow line) vs Q4 (the reference, blue line) of animal-based protein food intake in 1147 French-speaking adults from Québec. **B.** Radar plot of the HEFI-2019 component scores comparing Quarter 4 (yellow line) vs Q1 (the reference, blue line) of plant-based protein food intake in 1147 French-speaking adults from Québec. Because each component score has a different scale (e.g., Beverages is scored on 10 points while Whole-grain foods is scored on 5 points), component scores were standardized to percentages. The means of the HEFI-2019 component scores are adjusted for sex and age. Higher HEFI-2019 component scores indicate greater adherence to key recommendations on healthy food choices in the CFG-2019. Quarters of animal-based and plant-based protein foods in this population were identified based on usual (i.e., long-term) intakes calculated using the National Cancer Institute’s multivariate method and standardized to 2500kcal. HEFI-2019, Healthy Eating Food Index-2019. Q, Quarters.

# Supplementary References

1. Canada H. 2019 Canada’s Food Guide food classification system classification of foods and beverages into categories [Internet]. 2022 [cité 3 nov 2022]. Disponible sur: https://www.canada.ca/en/health-canada/services/publications/food-nutrition/2019-canada-food-guide-food-classification-system-foods-beverages-categories.html

2. Brassard D, Elvidge Munene LA, St Pierre S, Gonzalez A, Guenther PM, Jessri M, et al. Evaluation of the Healthy Eating Food Index (HEFI)-2019 measuring adherence to Canada’s Food Guide 2019 recommendations on healthy food choices. Appl Physiol Nutr Metab. 14 janv 2022;
